# The distribution of pain activity across the human neonatal brain is sex dependent

**DOI:** 10.1016/j.neuroimage.2018.05.030

**Published:** 2018-09

**Authors:** Madeleine Verriotis, Laura Jones, Kimberley Whitehead, Maria Laudiano-Dray, Ismini Panayotidis, Hemani Patel, Judith Meek, Lorenzo Fabrizi, Maria Fitzgerald

**Affiliations:** a Department of Neuroscience, Physiology, and Pharmacology, University College London, London, WC1E6BT, United Kingdom; b Elizabeth Garrett Anderson Obstetric Wing, University College London Hospitals, London, WC1E6DB, United Kingdom

**Keywords:** Pain, EEG, Nociception, Sex, Neonatal, Brain

## Abstract

In adults, there are differences between male and female structural and functional brain connectivity, specifically for those regions involved in pain processing. This may partly explain the observed sex differences in pain sensitivity, tolerance, and inhibitory control, and in the development of chronic pain. However, it is not known if these differences exist from birth. Cortical activity in response to a painful stimulus can be observed in the human neonatal brain, but this nociceptive activity continues to develop in the postnatal period and is qualitatively different from that of adults, partly due to the considerable cortical maturation during this time. This research aimed to investigate the effects of sex and prematurity on the magnitude and spatial distribution pattern of the long-latency nociceptive event-related potential (nERP) using electroencephalography (EEG). We measured the cortical response time-locked to a clinically required heel lance in 81 neonates born between 29 and 42 weeks gestational age (median postnatal age 4 days). The results show that heel lance results in a spatially widespread nERP response in the majority of newborns. Importantly, a widespread pattern is significantly more likely to occur in females, irrespective of gestational age at birth. This effect is not observed for the short latency somatosensory waveform in the same infants, indicating that it is selective for the nociceptive component of the response. These results suggest the early onset of a greater anatomical and functional connectivity reported in the adult female brain, and indicate the presence of pain-related sex differences from birth.

## Highlights

• Noxious stimulation causes widespread pain related potentials in the neonatal brain.
• This widespread pain response is more likely to occur in female babies.
• Brain responses to touch do not differ between male and female babies.
• Sex differences in human brain pain pathways are present from birth.

## Introduction

Although immature, the neonatal nociceptive system has the potential to encode painful stimuli (Fitzgerald, 2005; Verriotis et al., 2016a). Nociceptive activity can be detected in the human neonatal brain using several imaging techniques such as electroencephalography (EEG) (Fabrizi et al., 2011; Hartley et al., 2017; Jones et al., 2017; Slater et al., 2010c), near infra-red spectroscopy (NIRS) (Bartocci et al., 2006; Slater et al., 2006; Verriotis et al., 2016b), and functional magnetic resonance imaging (fMRI) (Goksan et al., 2015; Williams et al., 2015), providing insight into the neurophysiological development of nociceptive processing in the infant human cortex. Premature infants are more likely to exhibit non-specific bursting activity following tactile and noxious stimulation of the skin, which develops into modality specific event-related potentials (ERP) at around 35 weeks of age at time of study (Fabrizi et al., 2011). Nociceptive event-related potentials (nERP) are still not fully mature at term and are qualitatively different from those in adults, with neonates exhibiting different frequency patterns encoding noxious events (Fabrizi et al., 2016) and a more widespread fMRI blood oxygen level dependent (BOLD) response following pinprick stimulation (Goksan et al., 2015; Williams et al., 2015). Data from infant rodents has further demonstrated the postnatal maturation of cortical nociceptive processing, with nociceptive-evoked cortical activity changing in frequency content and power with age (Chang et al., 2016; Devonshire et al., 2015).

In adults, pain processing differs between males and females. Following experimental noxious stimulation, female subjects have higher sensitivity, lower tolerance, and reduced endogenous inhibitory control (Berkley et al., 2006; Fillingim et al., 2009; Kann et al., 2016; Popescu et al., 2010; Wiesenfeld-Hallin, 2005; for review, see Mogil, 2012). Recent studies suggest that some of the observed differences reflect greater sensory detection acuity in females, rather than a greater affective component of pain (Hashmi and Davis, 2014; Kann et al., 2016). Imaging studies have also reported sex differences in the magnitude of activation of certain brain regions following a noxious thermal stimulus (Derbyshire et al., 2002; Paulson et al., 1998). Specifically, while there is no difference in the pain report, female subjects exhibit larger ERPs following laser or contact-heat stimulation (Chao et al., 2007; Granovsky et al., 2016; Staikou et al., 2017). These differences may be partly explained by sex related dissimilarities in the brain structures underpinning pain processing. Males have a higher percentage of white matter and cerebrospinal fluid, excluding the corpus callosum (Cherney et al., 2008; Gur et al., 1999; Steinmetz et al., 1995), and females have a higher percentage of grey matter (Goldstein et al., 2001; Tomasi and Volkow, 2012). Females demonstrate more overall connectivity, primarily in the default mode network (Biswal et al., 2010; Bluhm et al., 2008; Filippi et al., 2013; Gong et al., 2009; Tomasi and Volkow, 2012), and show stronger interhemispheric connections while males show stronger intrahemispheric connections (Ingalhalikar et al., 2014). Laboratory studies indicate that there are also fundamental sex differences in the neuroimmune pathways underlying more prolonged pain states at spinal cord level (Mapplebeck et al., 2017).

It remains unclear whether these sex differences in pain processing are present from birth, or whether they emerge later in life. Previous studies have reported conflicting results with respect to sex differences in human infant pain behaviour following acute noxious stimulation (Grunau and Craig, 1987; Guinsburg et al., 2000; Holsti and Grunau, 2007; Owens and Todt, 1984), but it is important to note that infant behaviour does not always reflect brain activity (Jones et al., 2017). Resting state functional connectivity networks reveal that female children have stronger connectivity within the same network while males have stronger connectivity between different networks (Satterthwaite et al., 2015). Furthermore, the emergence of sex differences in later life can be influenced by neonatal factors: maternal cortisol significantly changes structural connectivity in females but not in males (Kim et al., 2017), and in rodents, sex differences in morphine analgesia can be reversed by neonatal hormone treatment (Krzanowska et al., 2002).

Since (i) some of the structural and functional brain networks related to pain processing are sexually dimorphic in adults and (ii) those same networks undergo significant development in the third trimester and postnatal period (e.g. Allievi et al., 2016; Doria et al., 2010; Hüppi et al., 1998; Kostović and Judaš, 2010; Malinger and Zakut, 1993; Seggie and Berry, 1972; Smyser et al., 2010; Volpe, 2009; for a review see Verriotis et al., 2016a), we hypothesised that significant sex differences in nociceptive processing may be detectable from birth. To test this, we have investigated the interaction between sex, gestational age at birth (GA) and the pattern of cortical nociceptive activity recorded with EEG following a clinically required heel lance in neonates born at 29–42 weeks gestation.

## Material and methods

### Participants

Participants were 81 newborn infants, no older than 2 weeks postnatal age (29–42 weeks GA^3^; 29–43 weeks of age at time of study), with the majority (75%) studied within six days from birth (Table 1). Babies were recruited from the postnatal, special care, or intensive care wards at the Elizabeth Garrett Anderson Obstetric Wing, University College London Hospital (UCLH) between 2007 and 2016. Babies were not eligible for inclusion in the study if they had congenital malformations or any abnormal cranial ultrasound scan, or were receiving analgesics at the time of study. All infants were clinically stable at the time of the study.

**Table 1:** Infant demographics.

Gestational age at birth (weeks)	36 (29–42)
Age at study (weeks)	37 (29–43)
PNA (days)	4 (0–13)
No. female	34 (42%)
No. babies stimulated on right foot	36
Birth weight (g)	2700 (1260–4592)
No. caesarean deliveries	34
Apgar score* @ 5 min	10 (6–10)

Values represent the median and range. Term = 37 weeks. PNA, postnatal age. *A simple and quick assessment, scored out of 10, to determine if a newborn requires any medical intervention immediately after birth.

Ethical approval was given by the NHS Health Research Authority. Informed written parental consent was obtained before each study. The study conformed to the standards set by the Declaration of Helsinki.

### Experimental protocol

Brain activity in response to a noxious procedure was monitored using electroencephalography (EEG). The noxious stimulus was a clinically required heel lance used for blood sampling performed by an experienced research nurse and time-locked to the ongoing EEG recording. A more detailed description of the lance procedure and EEG protocol has been published elsewhere (Slater et al., 2010c; Verriotis et al., 2016b).

### EEG recording

Recording electrodes (Ambu Neuroline disposable Ag/AgCl cup electrodes) were positioned individually by a clinical physiologist according to a modified international 10/10 electrode placement system at Fp1, Fp2, F3, F4, F7, F8, FCz, Cz, C3, C4, T7, T8, CPz, CP3, CP4, TP9, TP10, P7, P8, POz, O1, and O2. Although it was not always possible to apply the full set of electrodes, in 91% of the neonates at least 11 electrodes were used, and the Cz electrode (midline vertex) was used in all recordings (Inline Supplementary Table 1). The reference electrode was placed at either FCz or Fz, and all trials were re-referenced to Fz to allow comparison across trials. The ground electrode was placed on the chest, forehead, or at FC1/2. EEG activity was recorded from DC to ≥70 Hz, using the Neuroscan SynAmps2 EEG/EP recording system (Compumedics Neuroscan). A 50 Hz notch filter was used in 38 trials, and signals were digitised with a sampling rate of 2 kHz and a resolution of 24 bit.

### Event-related potential analysis

EEG traces were analysed using EEGLAB 10.2.5.8b (Swartz Center for Computational Neuroscience; Delorme and Makeig, 2004) and custom-written MATLAB scripts (MATLAB R2011b; The Mathworks, MA, USA). Traces were band-pass filtered between 1 and 30 Hz (2nd order bidirectional Butterworth filter), segmented into 1.7 s epochs starting from 0.6 s before the stimulus, and baseline-corrected using the pre-stimulus interval. EEG activity was inspected visually, and channels containing movement artefact (defined as activity exceeding ±100 μV),^4^ high-frequency muscle activity, or delta brush bursting activity (characterised by high voltage^5^ delta activity with over-riding alpha-beta oscillations), around the time of the nERP were removed (Inline Supplementary Fig. 1). Channels were rejected in only a third of trials across all age groups, and the proportion of channels retained per trial following rejections was similar across age groups (Inline Supplementary Table 2).

This study focused on the nociceptive-related ERP (nERP), which is a negative-positive waveform typically occurring within 400–700 ms post-stimulus (N3P3) (Fabrizi et al., 2011; Slater et al., 2010c, 2010b; Verriotis et al., 2016b). As a within-subject control, we also assessed the ERP preceding the nERP (N2P2, typically occurring within 200–400 ms post-stimulus), which is also evoked with tactile stimulation and is therefore usually considered a somatosensory response (Fabrizi et al., 2011; Slater et al., 2010c). Traces were aligned in order to correct for inter-subject latency jitter by Woody filtering (Bromm and Scharein, 1982; Woody, 1967) within 20–300ms and 350–700ms after stimulation, respectively, for the N2P2 and N3P3 ERPs (maximum jitter ±50 ms). For each trial, traces were aligned based on the Cz channel and the same alignment applied across all channels. Following Woody-filtering, traces were re-segmented into 1.5 s epochs starting from 0.5 s before the stimulus, and baseline-corrected between −0.5 and 0 s. There was one trial per subject resulting in 81 trials.

**Fig. 1:**
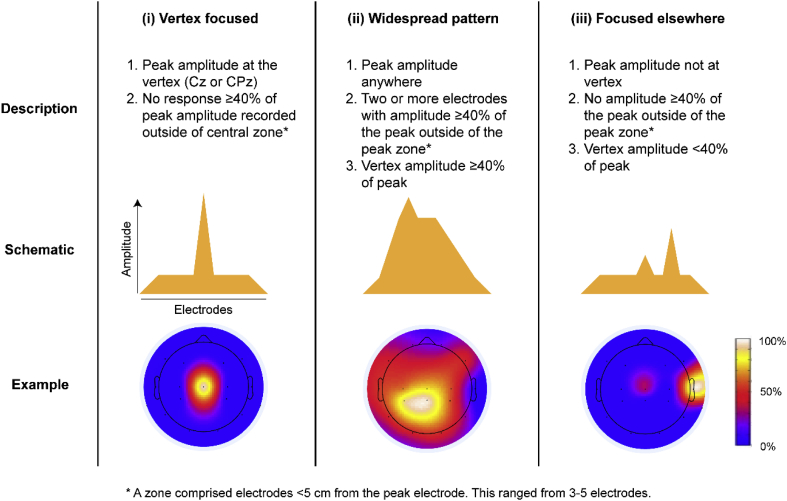
**Description and diagrammatic representation of three scalp ERP distribution patterns**: (i) focused at the vertex, (ii) widespread, and (iii) focused elsewhere. Typical examples of each pattern are shown as heat maps, where the amplitude of the N3P3 at each electrode is expressed as a percentage of the maximum amplitude within each individual subject. Note that only 4 trials were focused elsewhere and were subsequently removed from the analysis.

The incidence, amplitude, and scalp distribution of both waveforms, including the scalp location of the maximal amplitude, were recorded. Since both the N2P2 and N3P3 have been defined previously at vertex channels (Fabrizi et al., 2011; Slater et al., 2010c, 2010a; 2010b), the incidence of a response at these channels was determined, and considered present if recorded at either Cz or CPz (Inline Supplementary Methods). Trials with no response at either Cz or CPz were discarded from subsequent analysis. In the remaining trials, the vertex (Cz and CPz) response was used as a reference for peak detection at other electrodes to ensure consistency across all channels (Inline Supplementary Methods; Inline Supplementary Fig. 2). For each of the trials, all ERP amplitudes were calculated, by subtracting the N3 (or N2) amplitude from the P3 (or P2) amplitude, to identify the channel with the maximum ERP. The location of the maximal response was then categorised as either at the vertex electrodes, or at peri-central electrodes (C3, C4, CP3, CP4), or elsewhere. Finally, the scalp distribution of the N3P3 (and N2P2) for each included trial was classified, using the channel location of the maximal response and the distribution of response amplitudes at other channels, as: (i) focused at the vertex, (ii) widespread, or (iii) focused elsewhere (Fig. 1). The distribution was considered focused if channels distant (i.e. > 5 cm) from the maximal amplitude location were less than 40%^6^ of the maximal amplitude (which could be at any channel location), as described in Fig. 1. Trials with fewer than 7 electrodes after channel rejection were removed from this analysis.

### Statistical analysis

Statistical analysis was conducted in SPSS (IBM Corp.). The effect of sex on the incidence, amplitude and distribution (focused at the vertex/widespread^7^) of the nERP at the vertex, and the position and amplitude of the maximal response (vertex/peri-central/elsewhere) was tested with logistic (incidence, position and distribution; χ^2^ omnibus test) and linear (amplitude; F statistic from ANOVA model) regressions (using the ‘enter’ method). The same analysis was then repeated using gestational age at birth as the predictor variable. As EEG response amplitude is inversely related to the distance of the source from the scalp, it is plausible that a smaller response amplitude might be detected in infants with a larger head size as a result of a greater scalp thickness and distance from the source. Similarly, it may be more likely to detect an EEG response at a greater number of scalp locations in infants with a smaller head size. Therefore, for regressions including response amplitude and distribution we controlled for this using head circumference (measured at birth), which was correlated with GA (r (81) = 0.83, p < 0.001). The strong correlation between GA and head circumference (HC) at birth resulted in a degree of multicollinearity (VIF approximately equal to 3 for all regressions including GA and HC as predictors); therefore these regressions were re-run without HC to confirm that similar results were obtained. Similarly, for the distribution analysis, the maximal response amplitude was added as an additional confounding factor. Odds ratios (Exp(b) values) are reported for logistic regressions; these represent the increased likelihood of belonging to a test category vs. a reference category (e.g. likelihood of a maximal response at the vertex vs. elsewhere), with every increase in the predictor (e.g. a 1-week increase in GA). A summary of the sample sizes for these analyses is presented in Table 2.

**Table 2:** Sample sizes at each stage in the analysis.

Analysis	N
Incidence of nERP at the vertex	81
Amplitude of nERP at the vertex	69
Position of maximal response	69
Amplitude of the maximal response	69
Distribution pattern of response	67
Effect of sex and age on distribution	63

The incidence of a nERP at the vertex was assessed in the full sample (n = 81). Twelve babies without a response at the vertex were removed from subsequent analysis. Of the remaining 69 babies, 2 were removed because they did not have enough electrodes, after channel rejections, for assessment of their distribution pattern, and 4 were removed from the regression analysis because they were classified as having a “focused elsewhere” distribution pattern, which was too small a group for the regression analysis.

When head size data was not recorded, values (which were missing at random, MCRA test including head circumference at birth, gestational age at birth, and sex: χ^2^ (2) = 1.00, p = .606) were filled in using the maximisation estimation method (Dempster et al., 1977).

## Results

### Incidence and amplitude of the nERP at the vertex are not related to the sex of the subjects

**Fig. 2:**
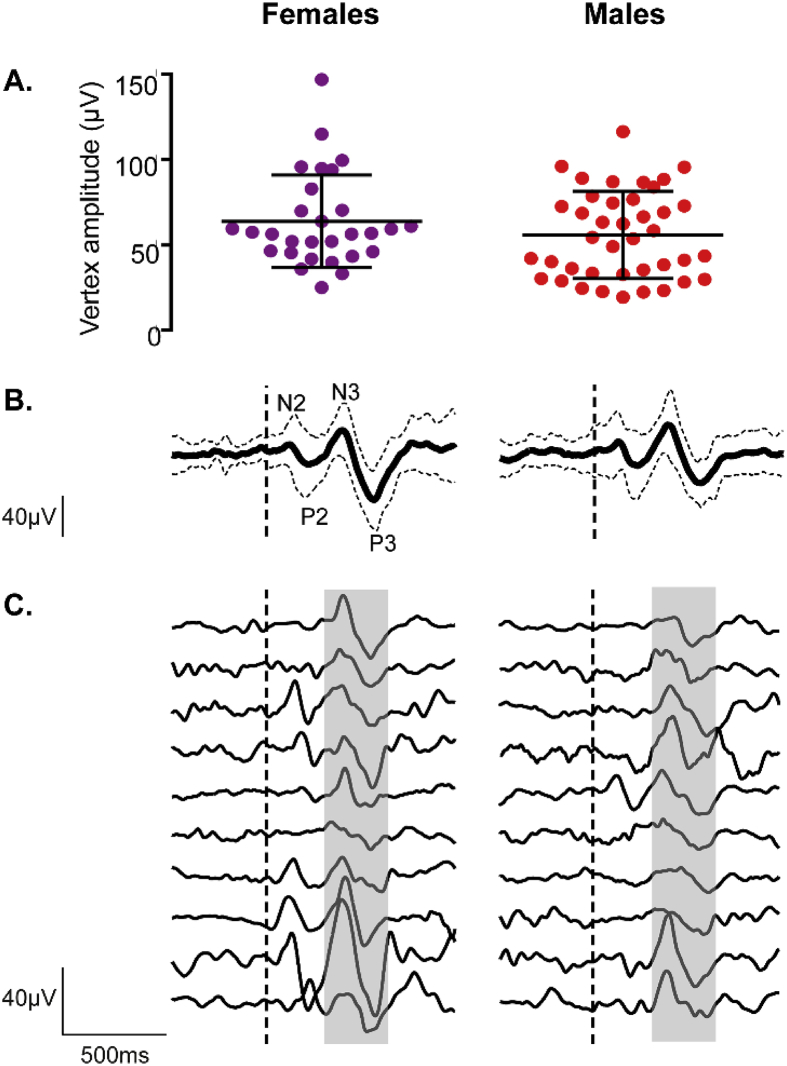
**Average N3P3 waveform and amplitudes for males and females.** A. N3P3 amplitude at the vertex (largest amplitude at Cz or CPz; average amplitudes at individual channels are presented in Inline Supplementary Fig. 4). Lines represent mean and standard deviation. B. Average N3P3 waveform at electrode Cz. C. Examples of 10 individual responses recorded at Cz. Grey shaded area highlights the typical latency window of the N3P3 (400–700ms). In B and C, the vertical dashed line marks the onset of the heel lance.

A nociceptive N3P3 response following a clinically required noxious heel lance was observed at the vertex (Cz or CPz) in 85.2% (69/81) of the trials. Fig. 2 illustrates this distinct nociceptive N3P3 waveform that follows the somatosensory N2P2. For the 69 trials with an N3P3 at the vertex, the mean vertex peak-to-peak amplitude was 59.2 ± 26.3 μV (mean ± SD), but there was considerable variation in individual N3P3 amplitudes (Fig. 2A) and morphology (Fig. 2C). The incidence of a response at the vertex and its amplitude were not related to the sex of the subjects (incidence: χ^2^ (1) = .001, p = .981; amplitude: F (2, 66) = 1.42, p = .250, OR = .14, p = .263). Similarly, the incidence and amplitude of the N3P3 response at the vertex were not significantly related to the gestational age at birth of the subjects (incidence: χ^2^ (1) = .35, p = .552; amplitude: F (2, 66) = .90, p = .412, OR = .11, p = .611), and clear responses could be identified even in the youngest babies (Inline Supplementary Fig. 3). Removing HC at birth as a predictor confirmed that GA was still not a significant predictor of the vertex N3P3 amplitude (Inline Supplementary Results).

### The maximal nociceptive cortical response is less likely to occur at the vertex in younger infants

**Fig. 3:**
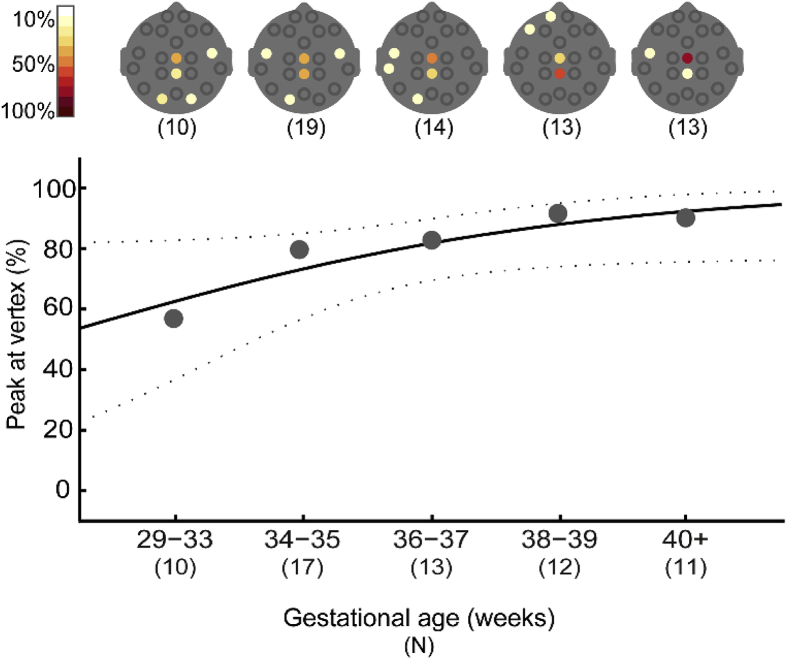
**Younger infants are less likely to have an N3P3 maximal at the vertex.** Top panel shows the proportion of babies in each age group that had a maximal N3P3 amplitude at each electrode (assessed in all trials in which a vertex response could be identified; n = 69). Bottom panel shows that the proportion of infants with peak N3P3 response at the vertex electrodes (compared to the “elsewhere” category) increases with gestational age at birth. Grey dots represent mean occurrence (calculated only for illustrative purposes); the black solid line is the logistic regression curve; and the dashed grey lines represent the 95% CI.

Although a nERP could be identified at Cz or CPz in 85% of trials, this response at the vertex was not always the maximal response. The maximal N3P3 response was located at the vertex electrodes in 73.9% (51/69) of trials, at the peri-central electrodes in 8.7% (6/69; C4, C3, CP4, CP3), and elsewhere in 17.4% (12/69). Sex was not a predictor of the location of the maximal response (Multinomial logistic regression with “elsewhere” as the reference category; likelihood ratio test: χ^2^ (2) = .52, p = .773). There was a trend for the location of the maximal response to depend on the gestational age at birth of the subjects, as younger infants were less likely to have a peak at the vertex and peri-central electrodes compared to older infants (vertex vs “elsewhere”: Odds ratio (OR) = 1.28,^8^ p = .055; peri-central vs. “elsewhere”: OR = 1.47, p = .062; Fig. 3), however the overall model was not significant (χ^2^ (2) = 5.19, p = .075). The amplitude of the maximal response was not related to the sex or age of the subjects (sex: F (2, 65) = 2.65, p = .078, OR = .13, p = .290; age: F (2, 65) = 2.23, p = .116, OR = −.12, p = .561). Removing HC at birth from the model resulted in a significant relationship between GA and maximal amplitude (Inline Supplementary Results). Similarly, the maximal somatosensory N2P2 response was also less likely to occur at the vertex in younger infants than in older infants (age: χ^2^ (2) = 14.36, p = .001, vertex vs “elsewhere”: OR = 1.51, p = .001; peri-central vs. “elsewhere”: OR = 1.26, p = .145).

### A widespread nociceptive cortical response is more likely to occur in females

**Fig. 4:**
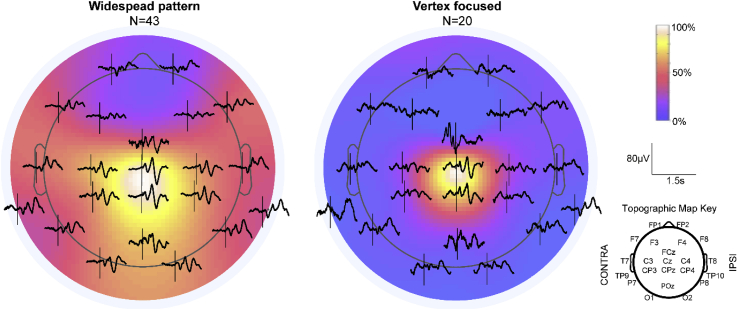
**Group average responses for widespread and vertex focused trials.** Heat maps showing the average distribution of the N3P3 response across the scalp for the widespread and vertex focused trials. The average amplitude at each electrode has been normalised to the groups' maximum response (Cz). Average waveforms at each electrode position are overlaid onto the heat maps.

**Fig. 5:**
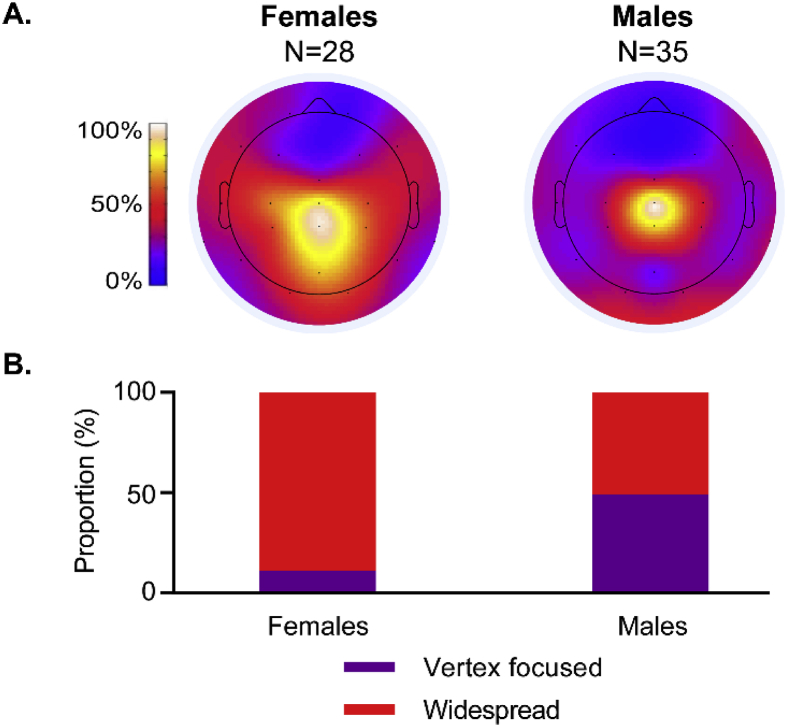
**Females are more likely to have a widespread response.** A. Average normalised topographic distribution of the nociceptive N3P3 peak-to-peak amplitude for females and males. B. The percentage of infants with a widespread vs. vertex focused N3P3 response, plotted separately for females and males.

We next explored the spatial distribution of the nERP across the scalp. We found that although the nERP could be identified at the vertex in 85% of trials and was maximal at these electrodes in 74% of trials, it was not always focused at this location (see Fig. 1 for definition of vertex focused). In fact, the nERP was focused at the vertex in only 40% (20/50) of those trials in which the vertex response was the maximal response on the scalp. Indeed, the response was more often widespread across the scalp (43/67, 64%) than focused (Fig. 4). This was significantly more likely in females, with 89% (25/28) of the females having a widespread distribution vs only 51% (18/35) of the males (χ^2^ (3) = 13.61, p = .003, OR = 7.52, p = .004; Fig. 5). The odds ratio indicates that females were 7.52 times more likely than males to exhibit a widespread N3P3 response. There was no effect of gestational age at birth on the proportion of widespread vs vertex focused responses (χ^2^ (3) = 5.67, p = .129, OR = .76, p = .149). Removing HC from this analysis confirmed that there was still no effect of GA on the response distribution (Inline Supplementary Results). In contrast, the distribution of the somatosensory N2P2 was not related to the sex or age of the infants (sex: χ^2^ (3) = 3.09, p = .378, OR = .67, p = .569; age: χ^2^ (3) = 5.01, p = .171, OR = .71, p = .133).

In light of the effects of age and sex on the position of the maximal response and distribution pattern, respectively, we also investigated any age and sex interaction on the maximal response position and on the distribution pattern using separate regressions for males and females, with GA as a predictor. We did not observe an interaction between the dependent variables.

## Discussion

Here we have shown that there are already significant sex differences in the distribution of nociceptive cortical event related activity in newborn infants, both preterm and full-term.

Most neonates had a maximal nERP at the vertex, but this is less likely for neonates born at a younger gestational age. Moreover, we found that this maximal response is not always narrowly focused at the vertex, but rather 64% of infants exhibit a significant response across several electrodes. Importantly, females are more likely to exhibit such a widespread pattern compared to males, independent of their gestational age at birth or head size, an effect which is selective for the nERP, the nociceptive component of the ERP response. With limited data on sex differences in the neonatal literature, this finding provides a significant contribution to the ongoing debate about the mechanisms underlying sex differences and pain (Fillingim et al., 2009).

### Noxious stimulation evokes widespread activity in neonates

The fact that the nociceptive response was widespread in most of the subjects has not been reported previously as earlier infant nERP analyses have been restricted to the vertex response (Fabrizi et al., 2011; Hartley et al., 2017; Jones et al., 2017; Slater et al., 2010c; Verriotis et al., 2015). The N3P3 response following the heel lance could be recorded from several electrodes across the scalp, and these responses, although smaller in amplitude than the response at the vertex electrodes, are not negligible, with the response at 60% of electrodes exceeding 20 μV (see Inline Supplementary Fig. 4). Widespread activity is consistent with the current understanding of a pain connectome, comprising several distributed but inter-connected brain regions (Davis et al., 2015; Kucyi and Davis, 2015; Mano and Seymour, 2015; Woo et al., 2015). A pin-prick stimulus evokes BOLD activity in a complex distributed brain network in neonates, which is more widespread than in adults (Goksan et al., 2015). This is consistent with our finding that nociceptive stimulation results in a response that, although maximal at the vertex, extends well beyond these electrodes in most of the infants.

### A widespread nociceptive response is more common in females

Females are more likely to have a widespread response than males. This may reflect the greater overall anatomical and functional connectivity and more efficient network organisation reported in the adult female brain (Gong et al., 2009; Tomasi and Volkow, 2012). As well as greater overall connectivity, female subjects are reported to have greater cortical activation following noxious stimulation. A positron emission tomography (PET) study using thermal noxious stimulation found that both sexes had activation of bilateral premotor cortex and several contralateral structures, but females had significantly greater activation in the contralateral prefrontal cortex, insula, and thalamus (Paulson et al., 1998). Thus, a more widespread cortical response in females in our study may suggest greater cortical involvement or connectivity following a noxious stimulus.

The greater activation and connectivity in female brains, both overall and following noxious stimulation, may underlie the differences between males and females in pain perception and self-report. Females are more sensitive to certain noxious stimuli (Berkley et al., 2006; Fillingim et al., 2009; Greenspan et al., 2007; Mogil, 2012), or exhibit greater sensory discrimination in response to acute or dynamic painful stimuli (Hashmi and Davis, 2014). As well as sex differences in experimental conditions, women are more likely to develop chronic pain conditions, and report more severe, frequent, and persistent pain (Fillingim et al., 2009; Mogil, 2012; Unruh, 1996). While there are likely hormonal and psychosocial mechanisms underlying these differences, our data points to fundamental biological differences in the development of cortical pain processing from birth.

Previous behavioural studies have produced conflicting results for the existence of pain-related sex differences in neonates. Following a noxious stimulus, Grunau et al. found that males have shorter latency time to cry and onset of facial changes compared to females (Grunau and Craig, 1987), while Guinsburg et al. reported greater pain related facial reactions in females, but no differences when using an alternative behavioural measure (Guinsburg et al., 2000). Others have reported no sex differences in infant pain behaviour (Holsti and Grunau, 2007; Owens and Todt, 1984; Stevens et al., 1994). However, behavioural responses are not always reflective of the cortical response following a noxious stimulus; indeed, neonates with higher levels of stress exhibit a dissociation between their behavioural and cortical responses (Jones et al., 2017). The lack of sex and age interactions in our study suggest that sex differences in the distribution of the nERP are present even in premature neonates. Thus, we have shown that during the equivalent of the final trimester, when there is significant cortical development, sex differences in nociception can already be observed.

### Younger neonates are less likely to exhibit a maximal response at the vertex

Previous research indicates that noxious heel lance elicits a response that is maximal at the vertex (Fabrizi et al., 2011; Slater et al., 2010b, 2010c). The present results show that while at a group level the nERP does peak at the vertex (Inline Supplementary Fig. 4B), consistent with previous reports, at the individual level, this is not always the case: the nERP peaked elsewhere in 26% of trials. Furthermore, the incidence of a maximal response at the vertex is even lower in babies born between 29 and 33 weeks gestation (57%), rising to 90% by term age (Fig. 3), a result that approached significance (vertex vs. ‘elsewhere’; p = .055).

Potentials that peak at midline electrodes are associated with bilateral cortical sources, with activation in both hemispheres summating at the midline (Scherg and Von Cramon, 1985). This is consistent with the view that pain processing results from the bilateral activation of several cortical and sub cortical areas (Davis et al., 2015; Kucyi and Davis, 2015; Mano and Seymour, 2015). As the nERP is maximal at the vertex by term age in most neonates, the apparent developmental trajectory of this response could reflect the development of structural and functional inter-hemispheric connections that mature from 35 weeks to term (Allievi et al., 2016; Doria et al., 2010; Kostović and Judaš, 2010; Smyser et al., 2010; Volpe, 2009; for a review, see Verriotis et al., 2016a). Indeed, the corpus callosum develops within this period and postnatally, facilitating the development of interhemispheric communication (Hüppi et al., 1998; Malinger and Zakut, 1993; Seggie and Berry, 1972). Alternatively, the maturation of the peak N3P3 at the vertex may reflect the developmental refinement of somatotopic organisation in the SI cortex, as the region representing the foot (the site of noxious stimulation in our study), is located at the top of the postcentral gyrus within the medial longitudinal fissure (Akselrod et al., 2017). Further research will be needed to confirm the developmental trajectory in a sample including a greater number of very preterm babies.

### Maximal N3P3 responses outside of the vertex

Some trials exhibited a peak response at peri-central electrodes rather than the midline vertex. It is possible that these non-topographically organised responses reflect normal individual variability, particularly as many cortical structures are still maturing (Allievi et al., 2016). Alternatively, a maximal response outside of the vertex area (including both “pericentral” (8.7%) and “elsewhere” (17.4%) locations) may be the result of evoked synchronisation of background bursting activity other than the typical delta brush pattern, which was specifically excluded in the present study, following the lance. We have previously shown that oscillations in the delta, alpha, beta, and gamma range can be synchronised by noxious stimulation in newborn infants (Fabrizi et al., 2016). Spontaneous bursting is a common feature of the newborn brain (André et al., 2010), and stimulation can increase the probability of this bursting, which is not necessarily located within the corresponding sensory cortex. For example, non-specific delta brush bursting activity, characterised by high voltage delta activity with over-riding alpha-beta oscillations, may be found within the temporal region following a noxious stimulus (Fabrizi et al., 2011). In general, spontaneous activity occurs in the peri-central or temporal-occipital regions, and disappears by term age (André et al., 2010; Tolonen et al., 2007; Watanabe and Iwase, 1972; for review see Whitehead et al., 2017), which is in line with our data showing that the peak response is less likely to be outside the vertex or peri-central region by term age.

### Incidence of a vertex response in preterm infants

The incidence of a vertex response was not associated with gestational age at birth in the present study. Previous research from our own group and others has reported an age-dependent shift from non-specific delta brush responses to nociceptive-specific event-related potentials (Fabrizi et al., 2011), with very premature babies (<32 weeks of age at time of study) being unlikely to exhibit a nERP (Hartley et al., 2016). However, very few babies in the present study were less than 32 weeks GA (n = 4), and only one of these were studied at less than 32 weeks of age at the time of study, making it difficult to compare with previous studies. A nERP could be identified in all four of these babies (age at study: 30–33 weeks). Moreover, these previous studies assessed the development of the nERP during the perinatal period using the term-age response as a reference point. Individual responses from term and preterm babies were compared to the average term baby response using Principal Component Analysis (PCA), which is sensitive to small differences in latency and morphology. In the present study, because we were investigating developmental changes in the nERP distribution across the scalp, it was more appropriate to use peak-to-peak analysis, which is less sensitive to differences in latency and morphology between age groups. The current results highlight that although immature relative to the term-age response (Fabrizi et al., 2011; Hartley et al., 2016), a nociceptive ERP can be identified in younger preterm babies.

### Methodological considerations

Two methodological considerations deserve mention. In the present study, we chose to control for head circumference at birth when assessing the effect of gestational age at birth on the amplitude and distribution of the responses, because we assumed that they might be modulated by head size. Unsurprisingly, GA and HC at birth were highly correlated, resulting in a degree of multicollinearity. Removing HC confirmed that results were consistent, except for the analysis of maximal response amplitude. Both GA and HC significantly predicted the amplitude of the maximal response across the scalp when tested on their own, but not when tested together. This highlights the importance of considering head circumference as a confounding factor when assessing developmental effects on response amplitude.

EEG studies exploring responses to sensory stimuli typically involve multiple repetitions per participant, allowing averaging within participants to increase the signal-to-noise ratio. This is not feasible in studies assessing cortical responses to noxious tissue-damaging stimuli in neonates, and the present results are necessarily based on single-trial data. However, noxious stimuli elicit large-amplitude responses in neonates (e.g. mean 59.2 ± 26.3 μV at the vertex in the present study) that are clearly discernible relative to the ongoing EEG within single trials. Furthermore, previous research has indicated that these are reproducible in babies requiring multiple procedures (Slater et al., 2010c; Verriotis et al., 2015), and consistent results have been reported across multiple studies in neonates (Fabrizi et al., 2011; Jones et al., 2017; Slater et al., 2010c, 2010a; 2010b; Verriotis et al., 2016b), young infants (Verriotis et al., 2015), and adults (Fabrizi et al., 2016, 2013). This is the first study to address individual variability in the topographical distribution of nociceptive-related cortical responses in neonates at different ages, and sex effects on cortical pain responses at birth.

### Conclusion

While neonatal nociceptive event related potentials elicited by a clinically required heel lance are often maximal at the vertex, the overall nERP response is more likely to be widespread across the scalp, rather than focused at the vertex. Moreover, females are more likely to exhibit a widespread response, independent of gestational age at birth. This may reflect greater activation or connectivity in female brains following noxious stimulation, from early development. As female adults are more sensitive to experimental pain, and are more likely to develop clinical pain conditions, this novel finding may reflect the presence of pain-related sex differences from birth.

## Author contributions

Conceptualization of study: M.F., L.F., J.M., M.V.; experimental design: M.F., L.F., M.V., J.M.; data collection: K.W., M.L-D., L.J., M.V.; data analysis: L.J., M.V., H.P., I.P.; data interpretation: L.J., M.V., L.F., M.F., J.M., K.W.; manuscript preparation: L.J., M.V.; discussion of results, critical comments and revision of manuscript: L.J., M.V., L.F., M.F., J.M., M.L-D., K.W.

## Appendix A Supplementary data

Supplementary Methods Supplementary Methods

Supplementary_Results Supplementary_Results

## Footnotes

3 GA refers to age at birth.

4 Head movements cause displacement of the electrodes, leading to a change in impedance between the electrodes and the scalp, affecting the voltage recorded. This kind of artefact is particularly common in neonates as they cannot be instructed to refrain from moving (Nevalainen et al., 2015; Pihko et al., 2004; White and Cooke, 1989). Threshold rejection of extreme voltage values is a typical technique to discard trials contaminated by gross movement artefact in ERP analysis (Luck, 2005). Threshold values are empirically chosen and can range from 20 to 100 μV (Gilmore et al., 1987; Luck, 2005). From experience (Jones et al., 2017; Verriotis et al., 2016b, 2015), a threshold of ±100 μV is sufficient for rejection of gross movement artefacts without data loss (this threshold is higher than the typical amplitude of a nERP).

5 Typically ranging between 50 and 300 μV (Whitehead et al., 2017).

6 This threshold was calculated by averaging the N3P3 amplitude across all channels in all trials and expressing this average amplitude (23 μV) as a percentage of the maximal amplitude across all trials (55 μV; 42%, rounded to 40%).

7 The category ‘focused elsewhere’ was excluded from this analysis due to too few trials classified as such.

8 This odds ratio can be interpreted as follows: the odds of a response at the vertex vs. elsewhere increases, on average, 1.28 times with every additional week of GA.
