# Supplementary material for: The distribution of pain activity across the human neonatal brain is sex dependent
Source: Neuroimage. 2018 Sep;178:69–77. doi: 10.1016/j.neuroimage.2018.05.030 (PMC6062722; doi:10.1016/j.neuroimage.2018.05.030)
Supplement: Supplementary_Fig [file mmc1.docx]

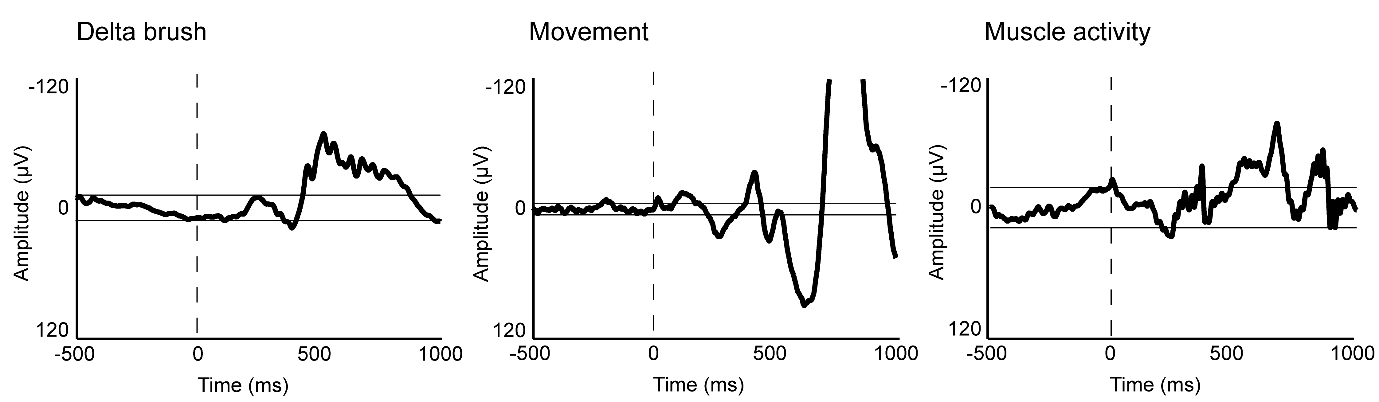


**Inline Supplementary Figure 1.** Examples of channels rejected due to contamination by delta brush activity (characterised by high voltage delta activity with over-riding alpha-beta oscillations), movement artefact (defined as activity exceeding ±100 µV), and high-frequency muscle activity around the time of the nERP. Dashed vertical line represents the time at which the stimulus was given; solid horizontal lines represent upper (+2 SD) and lower (-2 SD) thresholds.
