# Supplementary material for: The distribution of pain activity across the human neonatal brain is sex dependent
Source: Neuroimage. 2018 Sep;178:69–77. doi: 10.1016/j.neuroimage.2018.05.030 (PMC6062722; doi:10.1016/j.neuroimage.2018.05.030)
Supplement: Supplementary_Fig [file mmc2.docx]

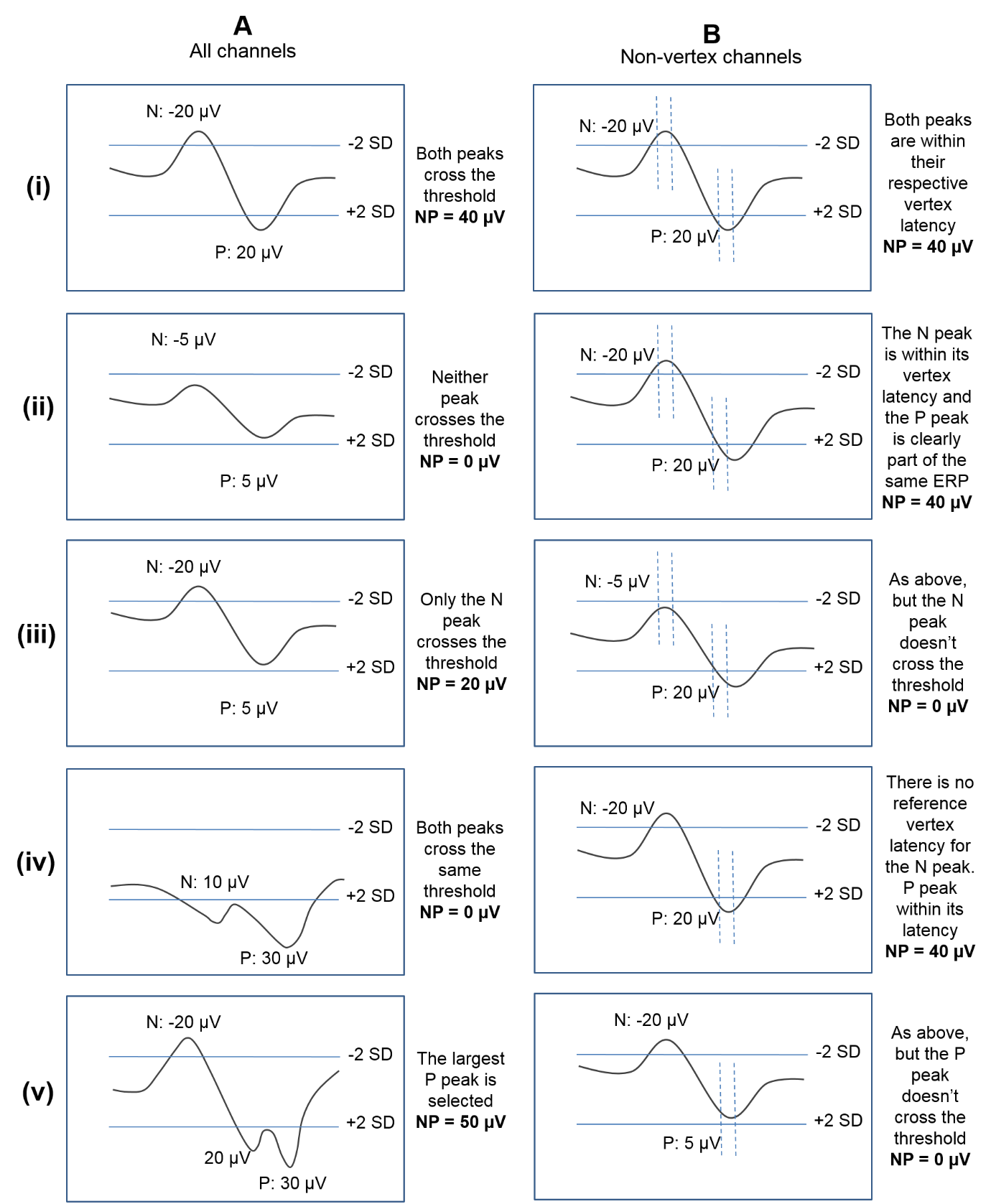


**Inline Supplementary Figure 2. Schematic describing peak selection criteria:** (A) for all channels, based on whether peaks crossed a threshold of ±2 standard deviations (SD) from the mean baseline period; and (B) for all non-vertex channels, based on their consistency with the vertex response in terms of latency. Solid horizontal lines represent upper (+2 SD) and lower (-2 SD) thresholds; dashed vertical lines represent a ±65ms reference window (from the combined vertex channel latency) within which non-vertex channel peaks had to be identified. N, negative peak; P, positive peak; NP, negative-positive peak-to-peak amplitude.
