# Supplementary material for: The distribution of pain activity across the human neonatal brain is sex dependent
Source: Neuroimage. 2018 Sep;178:69–77. doi: 10.1016/j.neuroimage.2018.05.030 (PMC6062722; doi:10.1016/j.neuroimage.2018.05.030)
Supplement: Supplementary_Fig [file mmc3.docx]

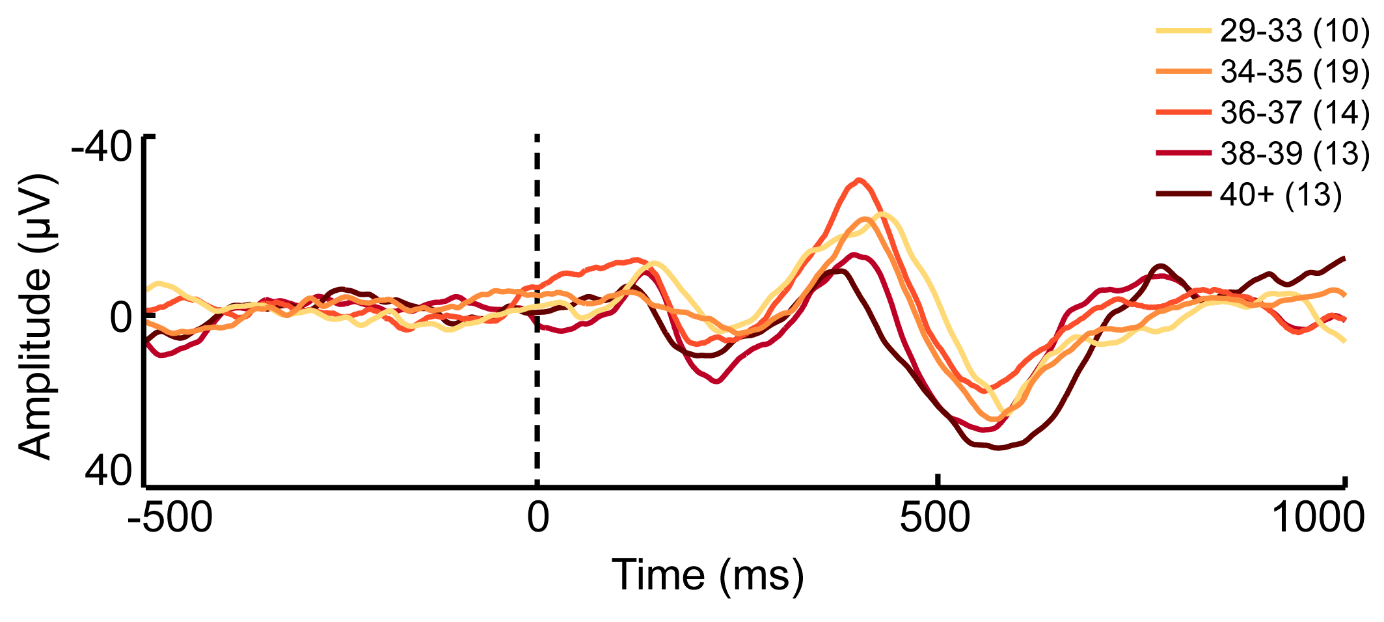


**Inline Supplementary Figure 3.** Average N3P3 response recorded at the Cz channel in all trials where the vertex response could be identified (n=69), plotted separately for each age group. Numbers in brackets indicate sample size within each age group.
