# Supplementary material for: The distribution of pain activity across the human neonatal brain is sex dependent
Source: Neuroimage. 2018 Sep;178:69–77. doi: 10.1016/j.neuroimage.2018.05.030 (PMC6062722; doi:10.1016/j.neuroimage.2018.05.030)
Supplement: Supplementary_Fig [file mmc4.docx]

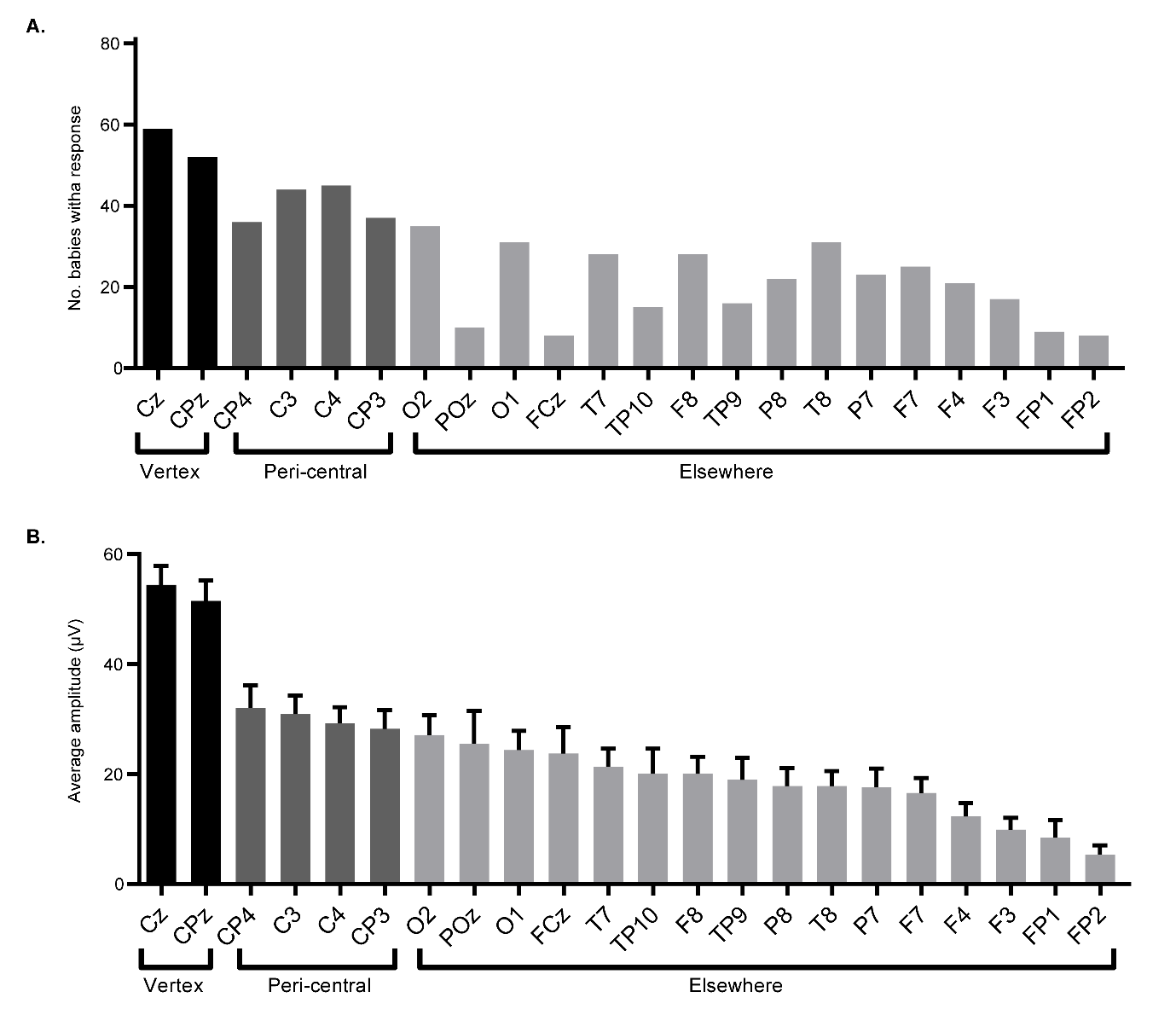


**Inline Supplementary Figure 4.** A) Total number of babies out of the sample of 81 babies that had an N3P3 for each electrode. Note that electrodes POz and FCz were recorded in only 24 and 8 trials, respectively. B) Average amplitude of the N3P3 at each electrode. Error bars represent standard deviation.
