# Supplementary material for: The distribution of pain activity across the human neonatal brain is sex dependent
Source: Neuroimage. 2018 Sep;178:69–77. doi: 10.1016/j.neuroimage.2018.05.030 (PMC6062722; doi:10.1016/j.neuroimage.2018.05.030)
Supplement: Supplementary_Table [file mmc5.docx]

**Inline Supplementary Table 1.**

Number of recording electrodes across all trials.

| **No. recording electrodes** | **N** |
| --- | --- |
| <7 | 1 |
| 7-9 | 1 |
| 10-12 | 7 |
| 13-15 | 3 |
| 16-18 | 55 |
| >18 | 14 |
