# Supplementary material for: The distribution of pain activity across the human neonatal brain is sex dependent
Source: Neuroimage. 2018 Sep;178:69–77. doi: 10.1016/j.neuroimage.2018.05.030 (PMC6062722; doi:10.1016/j.neuroimage.2018.05.030)
Supplement: Supplementary_Table [file mmc6.docx]

**Inline Supplementary Table 2**

Percentage of trials with rejected channels, and percentage of channels retained in these trials, across each age group.

| **Gestational age at birth (weeks)** | **% trials with rejected channels** | **Mean % channels retained in trials with rejected channels** |
| --- | --- | --- |
| <33 | 18 | 80 |
| 34-35 | 33 | 77 |
| 36-37 | 31 | 77 |
| 38-39 | 31 | 74 |
| 40+ | 35 | 76 |

Channels were rejected if they contained movement artefact, muscle activity, or delta brushes.
