# Supplementary material for: The distribution of pain activity across the human neonatal brain is sex dependent
Source: Neuroimage. 2018 Sep;178:69–77. doi: 10.1016/j.neuroimage.2018.05.030 (PMC6062722; doi:10.1016/j.neuroimage.2018.05.030)
Supplement: Supplementary Methods [file mmc7.docx]

**Inline Supplementary Methods**

*Peak detection criteria*

For all channels:

1. To determine whether peaks in a given trial were significantly different from baseline noise, we calculated the mean and standard deviation (SD) of the baseline period for each trial and used a threshold of ±2 SD from the mean baseline value. Peaks that did not cross this threshold were assigned a value of 0 (Inline Supplementary Figure 2Ai-iii).
2. In cases where the response drifted outside the ±2 SD threshold, such that both peaks crossed the same threshold, the peak-to-peak amplitude was set to 0 (Inline Supplementary Figure 2Aiv).
3. For traces with double peaks, the largest peak was chosen (Inline Supplementary Figure 2Av).

For all channels other than the vertex channels (Cz and CPz):

1. To ensure that we identified consistent responses across all channels, the combined vertex peak latencies (mean Cz and CPz latency for the N and P peaks) were used as a reference for peak selection. N and P peaks at other channels were identified within ± 65ms of the respective combined vertex peak latency (Inline Supplementary Figure 2Bi).
   1. If one peak was within this latency and the other was not, but was clearly part of the same biphasic ERP (with no other clear peaks in between), both peaks contributed to the peak-to-peak amplitude (Inline Supplementary Figure 2Bii).
   2. If one peak was outside the vertex latency and the other did not cross the threshold, the peak-to-peak amplitude was set to 0 (Inline Supplementary Figure 2Biii).
2. For trials in which there was no visible N or P peak at both Cz and CPz (thus no reference latency), peaks at other channels were identified based on the one remaining visible peak. For example, assuming that the P peak was identified at the vertex but the N peak was not:
   1. If the P peak was within the ±65ms window, and the N peak was clearly part of the same ERP, both peaks contributed to the peak-to-peak amplitude (Inline Supplementary Figure 2Biv).
   2. If the N peak but not the P peak could be identified, the peak-to-peak amplitude was set to 0 (Inline Supplementary Figure 2Bv).
