# Supplementary material for: The distribution of pain activity across the human neonatal brain is sex dependent
Source: Neuroimage. 2018 Sep;178:69–77. doi: 10.1016/j.neuroimage.2018.05.030 (PMC6062722; doi:10.1016/j.neuroimage.2018.05.030)
Supplement: Supplementary_Results [file mmc8.docx]

**Inline Supplementary Results**

*Assessing the effects of multicollinearity*

For regressions assessing the effect of GA on response amplitude and distribution, HC at birth was included as a confounding predictor. Since HC was highly correlated with GA, resulting in multicollinearity, these regressions were re-run without HC in the model. This confirmed that all results were unchanged after removal of the highly correlated variable, except for one (Supplementary Table 3).

**Supplementary Table 3**

Effect of removing HC at birth as a confounding predictor on the relationship between GA and response amplitude and distribution

| **Analysis** | **Effect of removing HC** | **Results** |
| --- | --- | --- |
| Vertex amplitude (Section 3.1) | No effect | F(1,67)=.53, p=.470, OR=-.09, p=.470 |
| Maximal amplitude (Section 3.2) | Significant* | F(1,66)=4.0, p=.049, OR=-.24, p=.049 |
| Response distribution (Section 3.3) | No effect | χ²(2)=4.23, p=.120, OR=.91, p=.369 |

*This shows that the amplitude of the maximal response decreases with increasing gestational age. It should be noted that when tested on its own, HC also significantly predicted maximal response amplitude (F(1,66)=4.16, p=.045, OR=-.24, p=.045).
